# Multigene Phylogenetics Reveals Temporal Diversification of Major African Malaria Vectors

**DOI:** 10.1371/journal.pone.0093580

**Published:** 2014-04-04

**Authors:** Maryam Kamali, Paul E. Marek, Ashley Peery, Christophe Antonio-Nkondjio, Cyrille Ndo, Zhijian Tu, Frederic Simard, Igor V. Sharakhov

**Affiliations:** 1 Department of Entomology, Virginia Polytechnic Institute and State University, Blacksburg, Virginia, United States of America; 2 Malaria Research Laboratory, OCEAC, Yaounde, Cameroon; 3 MIVEGEC (UMR IRD224-CNRS5290-UM1-UM2), Institut de Recherche pour le Développement (IRD), Montpellier, France; 4 Faculty of Medicine and Pharmaceutical Sciences, University of Douala, Douala, Cameroon; 5 Department of Biochemistry, Virginia Polytechnic Institute and State University, Blacksburg, Virginia, United States of America; International Atomic Energy Agency, Austria

## Abstract

The major vectors of malaria in sub-Saharan Africa belong to subgenus *Cellia*. Yet, phylogenetic relationships and temporal diversification among African mosquito species have not been unambiguously determined. Knowledge about vector evolutionary history is crucial for correct interpretation of genetic changes identified through comparative genomics analyses. In this study, we estimated a molecular phylogeny using 49 gene sequences for the African malaria vectors *An. gambiae*, *An. funestus*, *An. nili*, the Asian malaria mosquito *An. stephensi*, and the outgroup species *Culex quinquefasciatus* and *Aedes aegypti*. To infer the phylogeny, we identified orthologous sequences uniformly distributed approximately every 5 Mb in the five chromosomal arms. The sequences were aligned and the phylogenetic trees were inferred using maximum likelihood and neighbor-joining methods. Bayesian molecular dating using a relaxed log normal model was used to infer divergence times. Trees from individual genes agreed with each other, placing *An. nili* as a basal clade that diversified from the studied malaria mosquito species 47.6 million years ago (mya). Other African malaria vectors originated more recently, and independently acquired traits related to vectorial capacity. The lineage leading to *An. gambiae* diverged 30.4 mya, while the African vector *An. funestus* and the Asian vector *An. stephensi* were the most closely related sister taxa that split 20.8 mya. These results were supported by consistently high bootstrap values in concatenated phylogenetic trees generated individually for each chromosomal arm. Genome-wide multigene phylogenetic analysis is a useful approach for discerning historic relationships among malaria vectors, providing a framework for the correct interpretation of genomic changes across species, and comprehending the evolutionary origins of this ubiquitous and deadly insect-borne disease.

## Introduction

Malaria vectors belong to taxonomically diverse groups of anopheline mosquitoes. The genus *Anopheles* is divided into six subgenera including *Anopheles*, *Cellia*, *Kerteszia*, *Lophopodomyia*, *Nyssorhynchus* and *Stethomyia* [1]. Although the six *Anopheles* subgenera are monophyletic in origin, major malaria vectors do not form a monophyletic groupare polyphyletic [2]. Most malaria vectors are members of species complexes, which include both vectors and nonvectors [3], [4]. All major malaria vectors in Sub-saharan Africa belong to the subgenus *Cellia*, which consist of six series: Cellia, Neocellia, Myzomyia, Pyretophorus, Paramyzomyia, and Neomyzomyia. Previous analyses of the rDNA, and combined rDNA plus mtDNA data have supported the monophyly of a clade that includes Pyretophorus, Myzomyia, Neocellia and Neomyzomyia series [5]. *Anopheles gambiae* and *An. arabiensis* are major vectors of malaria in Africa and are members of the *An. gambiae* complex, which belongs to the series Pyretophorus. *Anopheles gambiae* consists of two molecular forms: the S form is widely distributed and the M form is restricted to West and Central Africa [6], [7]. A recent study has proposed to elevate the taxonomic status of the M form to a formal species level with the new name *Anopheles coluzzii* [8]. The *An. gambiae* complex also includes minor vectors (*An. bwambae, An. merus*, *An. melas*) and nonvectors (*An. quadriannulatus* and *An. amharicus*) [3], [8]. *Anopheles funestus* belongs to the Funestus subgroup, which is classified under the series Myzomyia and is divided in to five subgroups: Aconitus, Culicifacies, Funestus, Minimus, and Rivulorum [1]. *Anopheles moucheti* also belongs to the series Myzomyia. *Anopheles nili* is a member of the *An. nili* group that belongs to the series Neomyzomiya [1]. The Asian malaria mosquito *An. stephensi*, which is often used for phylogenetic comparisons with African mosquito species [5], [9]–[12], belongs to the series Neocellia within the subgenus *Cellia* [1].

Malaria in tropical humid savannas of Africa is quite stable with entomological inoculation rates (EIR: number of infective bites per person per year) varying between 50 and 300 [13]. *Anopheles gambiae* (M and S molecular forms), *An. arabiensis*, *An. funestus*, and *An. nili* are responsible for the majority of malaria cases in these areas [13]. These species, together with *An. moucheti*, a major vector in the equatorial forest of Central Africa, are responsible for >95% of the total malaria transmission on the African continent [14]. The habitat of these species varies considerably, and their contact with human habitation is of critical importance for addressing malarial transmission. *Anopheles gambiae*, *An. arabiensis*, and *An. funestus* breed in temporal or permanent freshwater pools. *Anopheles gambiae* is found mostly in humid savannas, *An. arabiensis* occupies arid savannas and steppes [15], while *An. funestus* has a continent-wide distribution in a broad variety of habitats [16]. *Anopheles nili* is as widely distributed as *An. gambiae* and is spread across most of West, Central, and East Africa, mainly in humid savannah and degraded rainforest areas [17], [18]. However, unlike the other major vectors, *An. nili* breeds in fast-moving streams and large lotic rivers exposed to light and containing vegetation or debris [13], [18]. A study of the ecological niche profiles of major malaria vectors in Cameroon demonstrated that the habitats of *An. gambiae*, *An. arabiensis*, and *An. funestus* have more overlap with each other than with the habitat of *An. nili* [17]. These results indicate a much more unusual geographic distribution of *An. nili*, and a different setting in which the species comes into contact with humans, thus revealing its pivotal role in malaria transmission in degraded, open-canopy forests in this region of Africa, and potentially elsewhere in the continent [18].

Knowledge about the phylogenetic relationships among the major African malaria vectors is essential for understanding the species-specific immune system responses from *Plasmodium falciparum* infection, and the accompanying evolutionary changes in the genomes of the mosquito species. However, it is still unknown if a particular lineage originated a long time ago or has emerged only recently. The diverse taxonomic positions of the major malaria vectors suggest that vectorial capacity evolved independently in all of these species. Each species group or complex, to which the major vectors belong, also includes nonvectors [1]. A phylogenetic reconstruction using mtDNA and rDNA has demonstrated a polyphyletic relationship among *An. arabiensis*, *An. gambiae*, *An. funestus*, *An. moucheti*, and *An. nili* [9]. However, stem-group relationships among the major African malaria vectors have not been unambiguously identified. For example, a Bayesian phylogenetic analysis using combined rDNA (18S and 28S) sequences, places the *An. gambiae* complex as sister to the remaining species of the subgenus *Cellia*, including *An. stephensi*, *An. funestus* and *An. nili*. However, a bootstrap consensus tree estimated with mtDNA sequences has been unable to provide strong support for these relationships due to a very low phylogenetic signal [9]. In contrast, other phylogenetic trees based on combined rDNA and mtDNA sequences have placed *Anopheles dirus* and *Anopheles farauti* (species from the same series as *An. nili*) as sister to the remaining species of the subgenus *Cellia*, including *An. gambiae*, *An. funestus* and *An. stephensi* [5].

An alternative approach to inferring phylogenetic relationships among species is a multigene phylogenetic analysis, which with the availability of many novel genetic markers and next-generation sequencing, has been successfully performed in many organisms. For example, 78 protein-coding genes have been effectively used to reconstruct a multigene phylogenetic tree of Choanozoa (unicellular protozoan phylum) and their evolutionary relationships to animals and fungi [19]. In another study, a phylogenetic tree based on 22 gene segments in Mustelidae, carnivorous mammals in the weasel family, has been inferred, and provides good support for both deep and shallow nodes in the group [20]. Importantly, multigene phylogenetics based on numerous concatenated gene sequences provides greater resolution and support, and is able to accurately reconstruct even ancient divergences, e.g. between animals and fungi [19]-[21]. According to a phylogenetic study of 106 orthologous genes from eight yeast species, the sufficient number of concatenated genes that are required to achieve the mean bootstrap value of 70% is three; while a minimum of 20 genes is required to recover >95% bootstrap values for each branch of the species tree [22]. Another study demonstrated an efficient phylogenetic approach by sampling and assembling transcriptomes of 10 mosquito species into data matrices containing hundreds of thousands of orthologous nucleotides from hundreds of genes [10]. However, that study did not include the major African malaria vectors except 3 species from the *An. gambiae* complex.

In our study, we investigated the phylogenetic relationships among major African malaria vectors as well as an Asian vector, *An. stephensi*. We selected 49 genes from the *An. gambiae* PEST strain genome [23], [24] distributed throughout 5 chromosomal arms. We identified orthologous sequences in the genomes of *An. nili* [25], *An. stephensi* [12], [26], *C. quinquefasciatus* [27], and *A. aegypti* [28], and in the transcriptome of *An. funestus* [29]. Phylogenetic trees were generated using a maximum likelihood (ML) and neighbor-joining (NJ) method from all genes individually, and with genes concatenated according to chromosomal arms. Results from different chromosomal arms were consistent with each other, and (1) placed *An. nili* as sister to the other African anopheline species, (2) indicated that the *An. gambiae* lineage split from the *An. funestus* and *An. stephensi* clade, and (3) African *An. funestus* and Asian *An. stephensi* were most closely related and most recently diverged taxa.

## Materials and Methods

### Genome-wide selection of genetic markers

**Figure 1:**
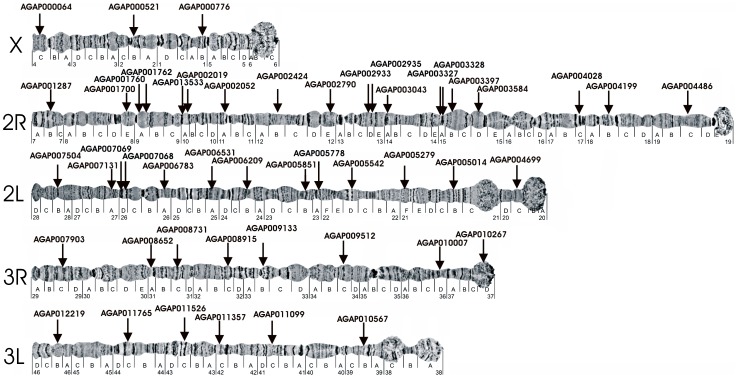
Distribution of genic phylogenetic markers in five chromosomal arms of *An. gambiae*. Names of the arms are placed near telomeres.

To ensure that gene trees provide multiple independent estimates of the species tree, it is important that genetic markers are distributed as uniformly as possible across the genome rather than clustered in a particular genomic region/single linkage group [30]–[32]. To resolve the molecular phylogeny of African malaria vectors, we selected 49 genes as molecular markers, widely distributed throughout the genome in all five chromosomal arms of the *An. gambiae* cytogenetic map [33] (Figure 1). In order to select genes evenly distributed throughout the genome, the AgamP3 genome assembly of the *An. gambiae* PEST strain (https://www.vectorbase.org/organisms/anopheles-gambiae/pest/agamp3) [24] was divided into 5 Mb segments. Genes were randomly selected within the 5 Mb segments of the *An. gambiae* genome with exon lengths between 364 and 1400 bp. Selected exons were transferred to Geneious 5.1.5 software (www.geneious.com) and used for finding homologous sequences in the *An. nili* genome assembly (DDBJ/EMBL/GenBank accession ATLZ00000000) [25] using Basic Local Alignment Search Tool (BLAST). The AnilD1 genome assembly of the *An. nili* Dinderesso strain is also available at VectorBase for BLAST and download (https://www.vectorbase.org/downloadinfo/anopheles-nili-dinderessocontigsanild1fagz). If no BLAST hits were present, another exon or gene was then selected. Appropriate exons from the *An. gambiae* PEST genome that had significant e-values (<1e-10) in BLAST searches against the *An. nili* genome were the candidate genes for further BLAST analysis. These exons were used to BLAST against the *An. gambiae* M5 (M form, Mali strain) (https://www.vectorbase.org/organisms/anopheles-gambiae/mali-nih-m-form/m5) and G4 (S form, Pimperena strain) (https://www.vectorbase.org/organisms/anopheles-gambiae/pimperena-s-form/g4) genome assemblies [34], the *An. stephensi* AsteI1 (DDBJ/EMBL/GenBank accession ALPR00000000, VectorBase: https://www.vectorbase.org/downloadinfo/anopheles-stephensi-indianscaffoldsastei1fagz) [12], [26], the *C. quinquefasciatus* (https://www.vectorbase.org/organisms/culex-quinquefasciatus/johannesburg/cpipj1) [27], and *A. aegypti* (https://www.vectorbase.org/organisms/aedes-aegypti/liverpool-lvp/aaegl1) [28] genome assemblies, as well as *An. funestus* transcriptome sequences (http://funcgen.vectorbase.org/annotated-transcriptome/Crawford_et_al_Anopheles_funestus) [29].

### Orthology detection

Two genes are orthologs if they diverged in a speciation event and are related by common ancestry [35]. To find orthologous genes, a Reciprocal Best Hits (RBH) method with an e-value threshold of at least 1e-10 was used [36]. In this method, orthologous pairs should have best reciprocal BLAST hits. In order to detect the RBH, *An. nili*, *An. stephensi*, *An. funestus*, *C. quinquefasciatus* and *A. aegypti* sequences were used to BLAST against the *An. gambiae* PEST strain, which has a mixture of sequences from M and S forms. Orthology was confirmed if reciprocal BLAST then finds the originally selected sequences in the *An. gambiae* PEST genome as the best hits.

### Gene alignment and phylogenetic analyses

Orthologous sequences with significant e-values in the BLAST search were then transferred to Molecular Evolutionary Genetics Analysis (MEGA 5.05) program [37]. The sequences were aligned using ClustalW alignment option in MEGA. Alignments were performed by adding the presumed most closely related species followed by the outgroup species. The sequence alignments are available upon request. Phylogenetic trees for each gene were constructed using a NJ method [38] in MEGA and a ML based method in RAxML version 7.5.3 [39]. A general time reversible (GTR) model with gamma distributed rate heterogeneity of nucleotide substitution (GTR + Γ) was used in the ML analysis. Support values for each clade were generated in RAxML by 1000 rapid bootstrap replicates. The 49 genes in 5 chromosomal arms were concatenated into a dataset of 42,300 bp and similarly analyzed using a RAxML tree search, while treating each gene as a separate unlinked partition. PhyloBayes version 3.3 was then used to estimate node divergence times under a relaxed clock log normal model [40], [41]. We used the best RAxML tree as a starting topology for PhyloBayes tree searches. Three fossils were used to calibrate the phylogeny: *Anopheles dominicanus*, 34 million years ago (mya) [42], *Culex winchesteri*, 34 mya [43], and *Paleoculicis minutus*, 70 mya, [44]. We assigned calibration fossils to the following nodes in the analysis: (1) *An. dominicanus* to the most recent node shared by *An. gambiae* and *An. nili*, (2) *C. winchesteri* to the most recent node shared by *C. quinquefasciatus*, and *Ae. aegypti*, and *P. minutus* to the most recent node shared by *An. nili* and *Ae. aegypti*. Placement of fossil calibrations were based on the global similarity or intuitive method that searches for the extant taxon that best corresponds to the fossil based on morphological similarity [45]. The best ML tree from the concatenated dataset was used as a rooted starting topology in the PhyloBayes analysis. Upon completion of the analysis, one fifth of the total chain length was discarded as burnin, and the posterior distribution of likelihoods, branch lengths, and rates were averaged and divergence dates summarized by the command: “readdiv”. Individual trees were visualized in FigTree version 1.4.0 [46] and consensus trees (those with a full complement of the 8 genomes) in DensiTree 2.0 [47].

## Results and Discussion

### DNA fragment length, alignment and matrix assembly

Based on the data obtained from VectorBase [48], the length of each chromosomal arm (in base pairs) is the following: X, 24393108; 2R, 61545105; 2L, 49364325; 3R, 53200684 and 3L, 41963435 (Table 1). In proportion to the length of each arm: 3, 19, 13, 8 and 6 genes were selected from X (Table S1), 2R (Table S2), 2L (Table S3), 3R (Table S4) and 3L (Table S5), respectively. Our sequencing data consisted of ≥364 bp-long gene fragments resulting in a total alignment of 41,124 bp based on the *An. gambiae* AgamP3 genome assembly. We obtained orthologous sequences of the selected genes from 8 genome assemblies representing 6 species. Support values for each clade were generated by 1000 bootstrap replicates and the reliability of nodes in the phylogenetic trees were assessed based on 70% as the cut-off value [49].

**Table 1:** Genome-wide distribution of genes used in the phylogenetic study.

Chromosome arm	Length (Mb)	Number of genes	Genes per 5 Mb
X	24.4	3	0.6
2R	61.5	19	1.5
2L	49.4	13	1.3
3R	53.2	8	0.8
3L	42.0	6	0.7
**Total**	**230.5**	**49**	**1.0**

### Phylogenetic relationships among African malaria vectors

Trees obtained using ML and NJ methods from individual genes agreed with each other, with a few exceptions. The most frequent branching pattern is: ((*Culex*, *Aedes*), (*An. nili*, ((*An. funestus*, *An. stephensi*), *An. gambiae*))). The phylogenies based on three X chromosome genes placed *An. nili* nested within other *Anopheles* vectors. Only in one of these trees with a high bootstrap value (>70%), was *An. nili* sister to the remaining malarial vectors and consistent with the dominant branching pattern (Figure S1, Figure S2). For the X chromosome trees, bootstrap values were generally low and branching pattern variable. Yet, the AGAP000064 gene tree recovers the common branching pattern mentioned above. For the 2R arm, 11 out of 19 in NJ phylogenetic trees and 8 out of 19 in ML phylogenetic trees recovered *An. nili* as sister with a high bootstrap value (Figure S3, Figure S2). For 2L arm, 8 NJ and 6 ML phylogenetic trees out of 13 recovered *An. nili* as sister to the other species of *Anopheles* (Figure S4, Figure S2). Eight phylogenetic trees were constructed for the 3R arm, of which 5 NJ and 3 ML trees had a high bootstrap value placing *An. nili* as sister to the ingroup species (Figure S5, Figure S2). Finally, 6 phylogenetic trees were constructed based on the genes located on the 3L chromosomal arm, of which 4 NJ and 4 ML trees had a high bootstrap value supporting the sister group placement of *An. nili* relative to other African vectors (Figure S6, Figure S2).

**Figure 2:**
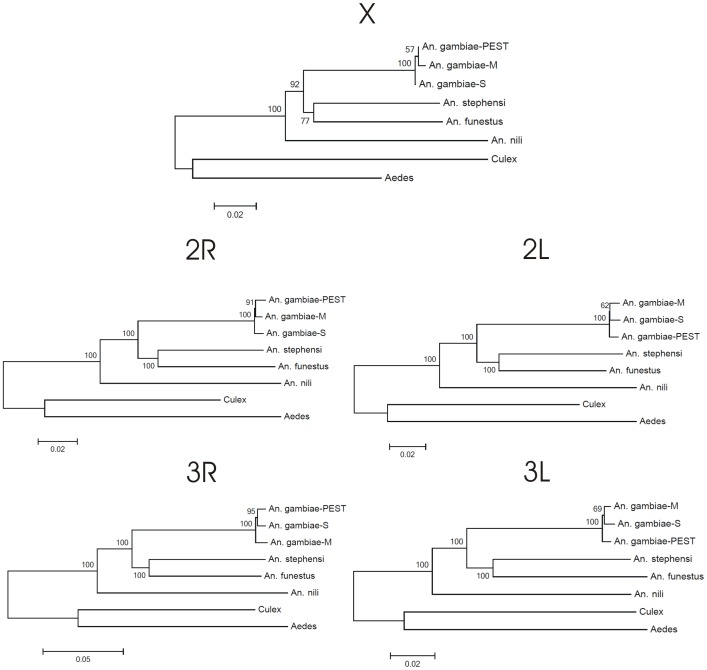
Phylogenetic NJ trees build from concatenated sequences located in 5 chromosomal arms. Bootstrap values are shown on branches of phylogenetic trees as percentages.

**Figure 3:**
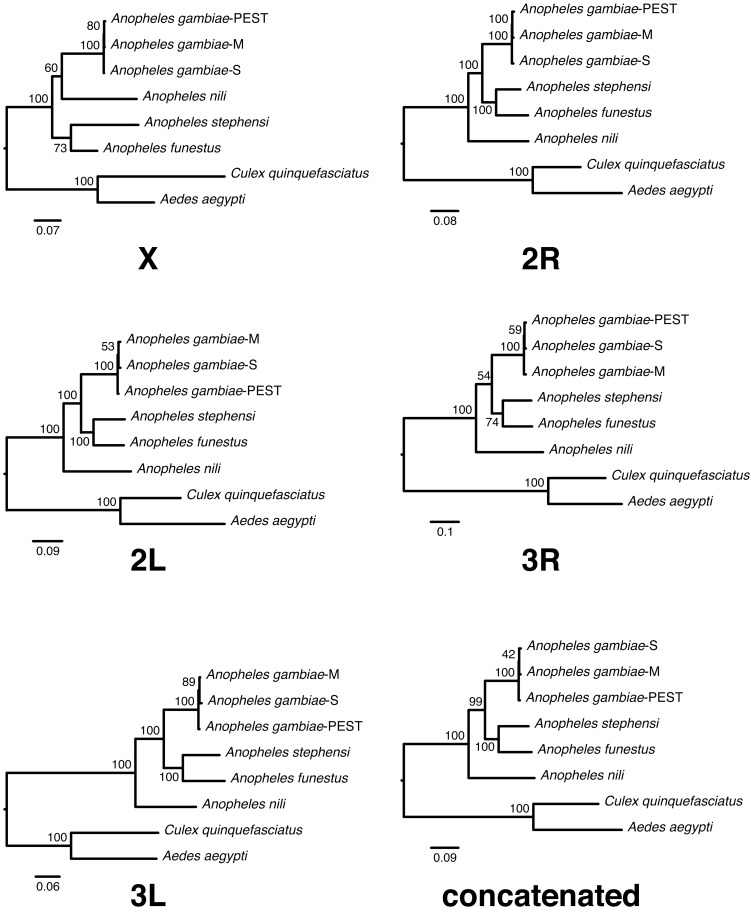
Phylogenetic ML trees build from concatenated sequences located in 5 chromosomal arms and the partitioned ML tree for all arms combined. Bootstrap values are shown on branches of phylogenetic trees as percentages.

Previous studies have shown that analysis of data sets of concatenated genes is useful for resolving species phylogenetic trees [22]. This is especially the case when independent gene trees converge on a dominant topology, as is shown in our data. We created trees using sequences for all genes concatenated according to the five chromosomal arms with NJ and ML methods (Figures 2, 3). In all trees, *A. aegypti* and *C. quinquefasciatus* were clustered separately as outgroup species. The NJ and ML trees inferred with concatenated genes provided consistent support that *An. nili* is sister to the rest of *Anopheles* species, except for the X chromosome ML tree where *An. nili* is swapped with *An. funestus* + *An. stephensi* (Figure 3). There is also consistent support for *An. funestus* clustered together with *An. stephensi*. The *Anopheles gambiae* branch, which itself comprises two rapidly diverged molecular forms (M and S) as well as the PEST reference strain, is consistently recovered as sister in relation to the *An. funestus* + *An. stephensi* clade.

**Figure 4:**
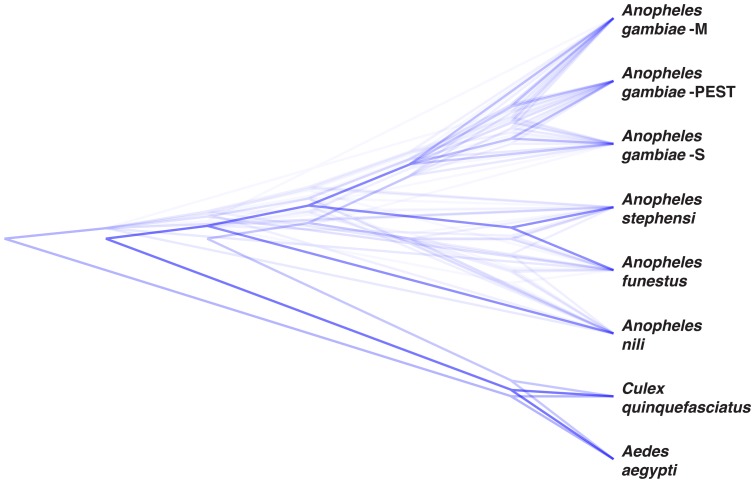
A consensus cladogram of the 49 gene trees obtained with the hierarchical clustering method implemented in DensiTree.

We attempted to compare our phylogenetic approach to other previously used methods. The only other phylogenetic study that included *An. gambiae*, *An. funestus*, *An. nili*, and *An. stephensi* used mtDNA and nuclear rDNA [9]. A parsimony bootstrap consensus tree based on the mtDNA sequence had a very low phylogenetic signal (resulting from highly variable COI and COII sequences) and, therefore, could not resolve the phylogeny with confidence. A Bayesian phylogenetic analysis using rDNA sequences placed the *An. gambiae* complex sister to the remaining species of *Cellia*, including *An. stephensi*, *An. funestus* and *An. nili* [9]. However, Marshall et al. 2005 included seven *Cellia* species not represented in our study. Although potentially related to taxon sampling and/or a smaller dataset, the trees—even with the seven additional taxa pruned—are in contradiction with our concatenated trees, which consistently show the branching pattern: (*An. nili*, ((*An. funestus*, *An. stephensi*), *An. gambiae*)). This dominant pattern was also recovered by using the consensus-based hierarchical clustering method in DensiTree (Figure 4). Interestingly, another phylogenetic study using combined rDNA and mtDNA sequences has demonstrated a sister group position of *An. dirus* and *An. farauti,* in relation to other species of the subgenus [5]. The Asian mosquitoes *An. dirus* and *An. farauti* together with the African mosquito *An. nili* all belong to the series Neomyzomiya, consistently suggesting that this series is a phylogenetically separate sister clade from the remaining series: Pyretophorus (*An. gambiae, An. arabiensis*), Myzomyia (*An. funestus, An. moucheti*) and Neocellia (*An. stephensi*) [1].

### Hypothesized evolutionary history of African malaria vectors

**Figure 5:**
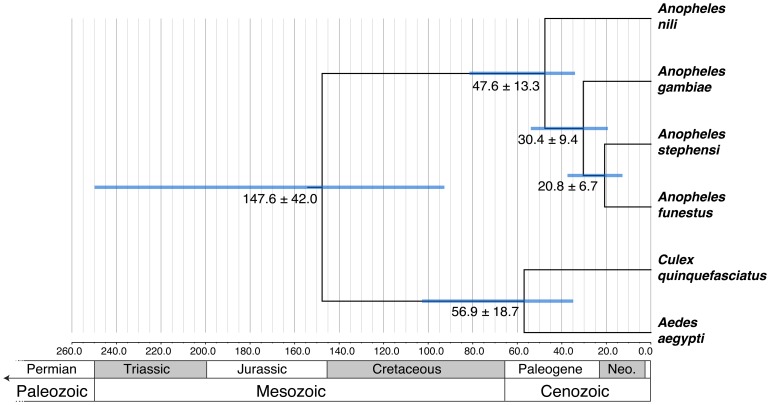
Time-calibrated tree and divergence dates estimated with PhyloBayes. Nodes are at mean divergence dates (in millions of years with standard errors). Blue bars indicate a minimum/maximum 95% confidence interval estimated from the post burnin parameter distribution. Geologic time scale derived from the Geological Society of America: http://www.geosociety.org/science/timescale/.

Our phylogenetic analysis of multiple genes indicates that the *An. nili* lineage split from the other African *Anopheles* species 47.6±13.3 mya (Figure 5). This date is congruent with the estimate of 43.1 mya of divergence between *An. gambiae* and *Anopheles atroparvus* in a prior study investigating divergence times in Culicidae [50]. A basal phylogenetic position of *An. nili* with respect to the other major malaria vectors indicates that traits relevant to increased vectorial capacity evolved in a convergent pattern. African vectors appear to have originated less frequently in the lineage to which *An. nili* belongs. (However, this appears to be the pattern for contemporary species as we do not yet know species extinction rates in the lineage, or differential losses and gains of vectorial capacity.) Four species had been described within the *An. nili* group based on morphological and genetic (isoenzymes and ribosomal DNA second internal transcribed spacer (rDNA ITS2) and D3 28S regions) differences: *An. nili s.s., Anopheles somalicus, Anopheles carnevalei*, and *Anopheles ovengensis* [51], [52]. These species, spare *An. nili*, show decreased vectorial capacity. A comprehensive study in Cameroon confirmed that *An. nili* is the only major malaria vector of the group and in contrast emphasized the exophagic behavior of *An. ovengensis* and *An. carnevalei* [53], [54]. *Anopheles nili* is mainly present in degraded forests, while *An. ovengensis* and *An. carnevalei* are found in the deep forests of Cameroon [18]. *Anopheles ovengensis* is a secondary vector, *An. carnevalei* is an inefficient vector, and *An. somalicus* is a nonvector because of its strong zoophilic and exophilic habits [54], [55]. In contrast, the clade that is sister to the *An. nili* lineage has produced several efficient African malaria vectors: *An. funestus*, *An. arabiensis*, *An. gambiae*, and, possibly, *An. moucheti*.

A recent study on the genetic structure of species of the *An. nili* group using a combination of microsatellites, rDNA and mitochondrial DNA markers, demonstrated unexpectedly high genetic divergence among new cryptic members of the *An. nili* group in Cameroon [56]. Also, a comparative cytogenetic analysis of polytene chromosomes revealed significant differences in banding pattern and structure of heterochromatin between *An. nili* and *An. ovengensis* [57]. This high genetic and chromosomal divergence within the *An. nili* group in central Africa suggests that the lineage originated and diversified in the region corresponding to the present day equatorial forest. At 55.0 mya, the Paleocene-Eocene Thermal Maximum was underway. At this time, there was extensive forest proliferation and simultaneous rapid diversification and increase in the abundance of mammals, including primates, even-toed ungulates, and horses [58]. The divergence of the *An. nili* group 47.6 mya from its sister group—*i.e*. the remaining African malaria vectors—corresponds in timing to this event (Figure 5). In contrast, the species and population diversity in the equatorial forest is very low for the *An. gambiae* complex and the *An. funestus* group.

Regardless of the possible region of origin in Africa, each of the major African malaria vectors seems to have a sister taxon among Asian malaria mosquito species. For example, a Bayesian phylogenetic analysis using rDNA sequences indicated that *An. nili* is a sister taxon with Asian *An. dirus* and *An. farauti* [9]. According to our phylogenetic tree, *An. funestus* and *An. stephensi* are closely related and more recently diversified (20.8±6.7 mya) (Figures 4, 5). Another study based on morphological characteristics as well as rDNA and mtDNA sequences considered Afrotropical Funestus and Afro-Oriental Minimus groups as sister taxa [9], [59]. However, the African continent was surrounded by water during the Neogene period (from 23.03±0.05 to 2.588 mya) making mosquito migrations between Africa and Asia unlikely. In our analysis, the *An. gambiae* lineage diverged 30.4±9.4 mya, but the members nested within the *An. gambiae* diversified much more recently (Figures 4, 5). Consistent with this Asian-African sister group trend, a clade composed of *An. gambiae* and *An. arabiensis* is a sister taxon with a clade composed of the Asian *Anopheles* species *subpictus* and *sundaicus* in a combined phylogenetic analysis of mtDNA and rDNA [9]. Moreover, the fixed 2La chromosomal inversion typical to *An. arabiensis* and *An. merus* was also found in two species from the Middle Eastern *An. subpictus* complex [60]. *Anopheles merus* was not sampled in our phylogeny, or in the phylogeny of Marshall et al. 2005 [9], but it was a sister to other members of the *An. gambiae* complex in the chromosomal phylogeny [12]. Therefore, the *An. gambiae* complex may be closely related to other Asian malaria vectors. Overall, these data suggest that mosquitoes from divergent lineages of the subgenus *Cellia* (*An. gambiae*, *An. arabiensis*, *An. funestus*, and *An. nili*) experienced potentially repeated migrations between Africa and Asia spanning the Cenozoic Era (from 66 mya to the present).

## Conclusion

Comparative genomic analyses of epidemiologically important traits will be more informative if performed within an accurate phylogenetic framework. Inferring the evolutionary history of African malaria vectors is crucial for establishing the association between evolutionary genomic changes with key features like the origin and loss of human blood choice, ecological and behavioral adaptations, and association with human habitats. A recent reconstruction of chromosomal phylogeny in the *An. gambiae* complex strongly suggests a repeated origin of increased vectorial capacity during evolution of African mosquitoes [12]. New genome assemblies for 16 species of *Anopheles* mosquitoes is a valuable resource for phylogenetic studies and comparative genomic analyses [61]. Although, the 16 species cluster includes vectors from different regions of the world, it lacks some of the major African malaria vectors, such as *An. nili.* Spanning diverse parts of the phylogenetic tree, African malaria vectors of subgenus *Cellia* represent a unique system for studying the evolution of vectorial capacity. Our study concludes that *An. nili* belongs to a basal lineage probably originating 47.6±13.3 mya. Other African malaria vectors originated more recently, and independently acquired traits related to vectorial capacity. This phylogeny will affect the interpretation of results from comparative genomics studies of malaria mosquito species. For example, genetic variation shared with *An. nili* might be considered ancestral polymorphism. We found strong agreement between gene trees reconstructed using multiple unlinked genes from distinct chromosomes indicating that next-generation sequence data are highly valuable for accurately inferring phylogenetic relationships among mosquito species, and providing an informative evolutionary context to understand the origins and maintenance of this pervasive and debilitating human disease.

## Supporting Information

Figure S1 **Phylogenetic NJ trees build for AGAP000064, AGAP000521, and AGAP000776 gene sequences located in the X chromosome.** Bootstrap values are shown on branches of phylogenetic trees as percentages.
(TIF)

Figure S2 **Phylogenetic ML trees build from sequences of 49 genes located in 5 chromosomal arms.** Bootstrap values are shown on branches of phylogenetic trees as percentages.
(PDF)

Figure S3 **Phylogenetic NJ trees build for AGAP001287, AGAP001700, AGAP002019, AGAP002252, AGAP002424, AGAP002790, AGAP003043, AGAP003397, AGAP003584, AGAP004028, AGAP004199, AGAP004486, AGAP001760, AGAP001762, AGAP002933, AGAP002935, AGAP013533, AGAP003327, and AGAP003328 gene sequences located in the 2R chromosomal arm.** Bootstrap values are shown on branches of phylogenetic trees as percentages.
(TIF)

Figure S4 **Phylogenetic NJ trees build for AGAP004699, AGAP005014, AGAP005279, AGAP005542, AGAP005851, AGAP006209, AGAP006531, AGAP006783, AGAP007131, AGAP007504, AGAP005778, AGAP007068, and AGAP007069 gene sequences located in the 2L chromosomal arm.** Bootstrap values are shown on branches of phylogenetic trees as percentages.
(TIF)

Figure S5 **Phylogenetic NJ trees build for AGAP007903, AGAP008652, AGAP008731, AGAP008915, AGAP009133, AGAP009512, AGAP010007, and AGAP010267 gene sequences located in the 3R chromosomal arm.** Bootstrap values are shown on branches of phylogenetic trees as percentages.
(TIF)

Figure S6 **Phylogenetic NJ trees build for AGAP010567, AGAP011099, AGAP011357, AGAP011526, AGAP011765, and AGAP012219 gene sequences located in the 3L chromosomal arm.** Bootstrap values are shown on branches of phylogenetic trees as percentages.
(TIF)

Table S1 **Selected genes from X chromosome and length of orthologous sequences in 6 species.**
(DOCX)

Table S2 **Selected genes from 2R chromosome and length of orthologous sequences in 6 species.**
(DOCX)

Table S3 **Selected genes from 2L chromosome and length of orthologous sequences in 6 species.**
(DOCX)

Table S4 **Selected genes from 3R chromosome and length of orthologous sequences in 6 species.**
(DOCX)

Table S5 **Selected genes from 3L chromosome and length of orthologous sequences in 6 species.**
(DOCX)
