# Supplementary figures and images for: Multigene Phylogenetics Reveals Temporal Diversification of Major African Malaria Vectors
Source: PLoS One. 2014 Apr 4;9(4):e93580. doi: 10.1371/journal.pone.0093580 (PMC3976319; doi:10.1371/journal.pone.0093580)

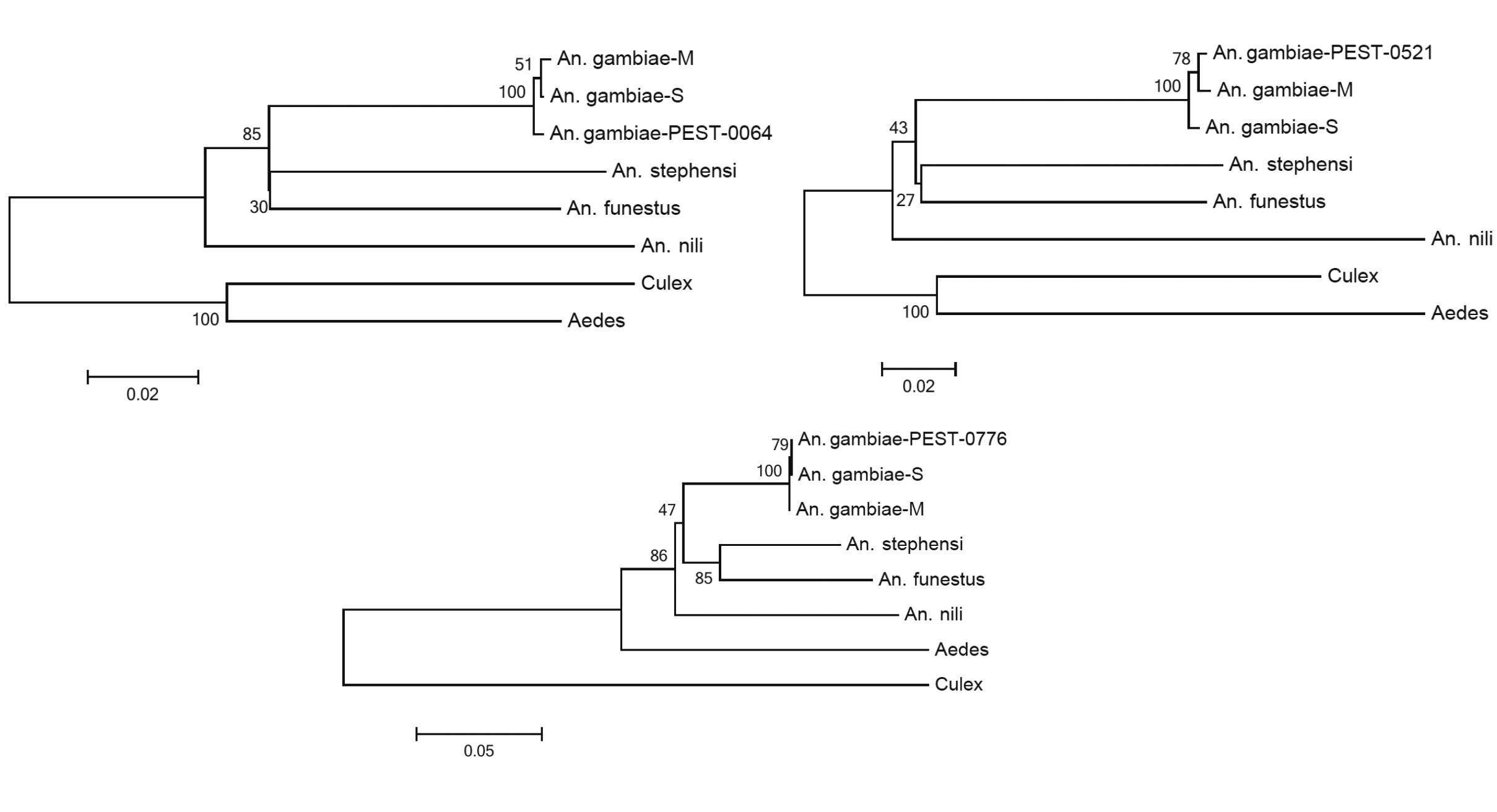

Supplement: Figure S1 — Phylogenetic NJ trees build for AGAP000064, AGAP000521, and AGAP000776 gene sequences located in the X chromosome. Bootstrap values are shown on branches of phylogenetic trees as percentages. (TIF) [file pone.0093580.s001.tif]

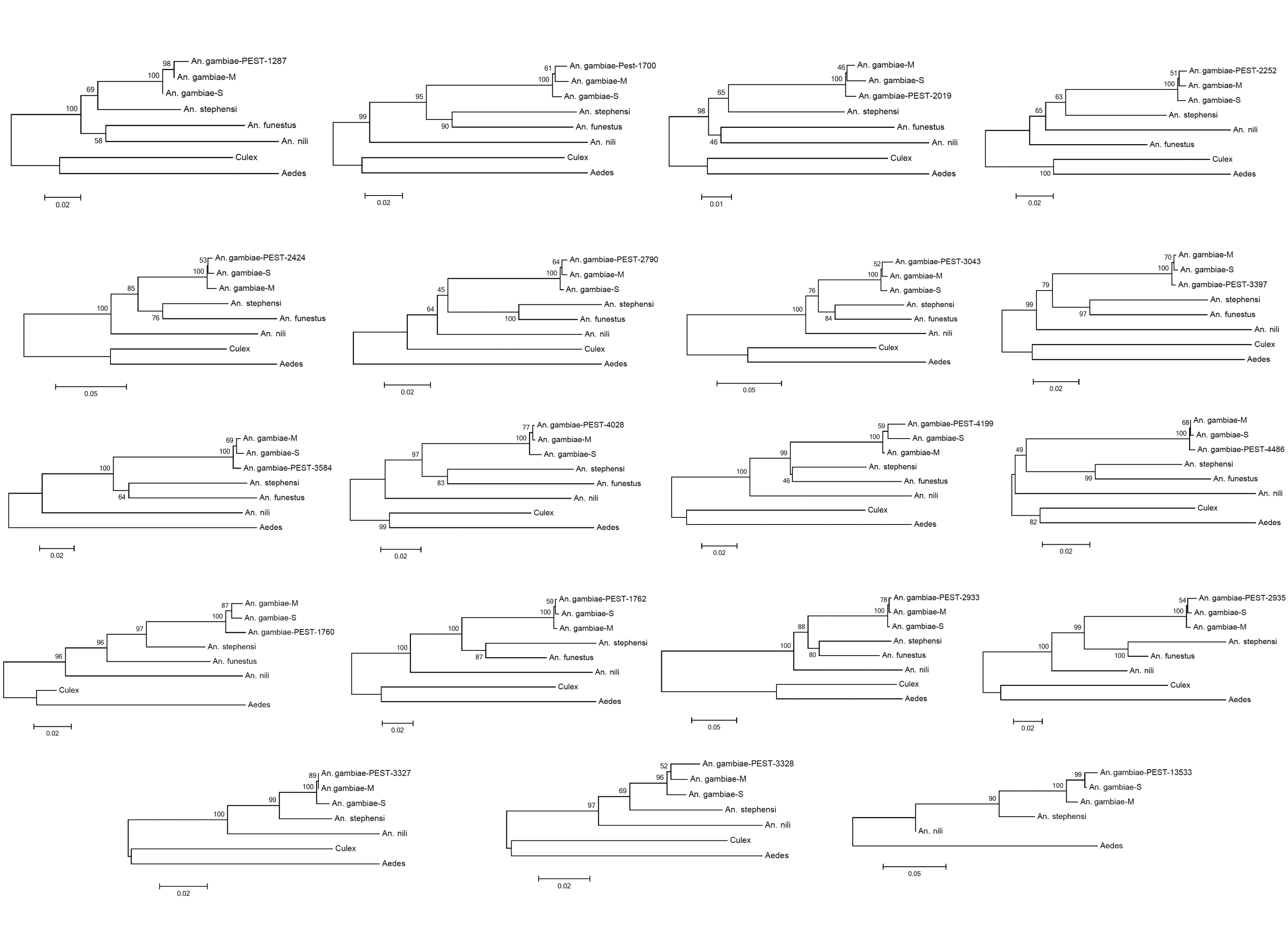

Supplement: Figure S3 — Phylogenetic NJ trees build for AGAP001287, AGAP001700, AGAP002019, AGAP002252, AGAP002424, AGAP002790, AGAP003043, AGAP003397, AGAP003584, AGAP004028, AGAP004199, AGAP004486, AGAP001760, AGAP001762, AGAP002933, AGAP002935, AGAP013533, AGAP003327, and AGAP003328 gene sequences located in the 2R chromosomal arm. Bootstrap values are shown on branches of phylogenetic trees as percentages. (TIF) [file pone.0093580.s003.tif]

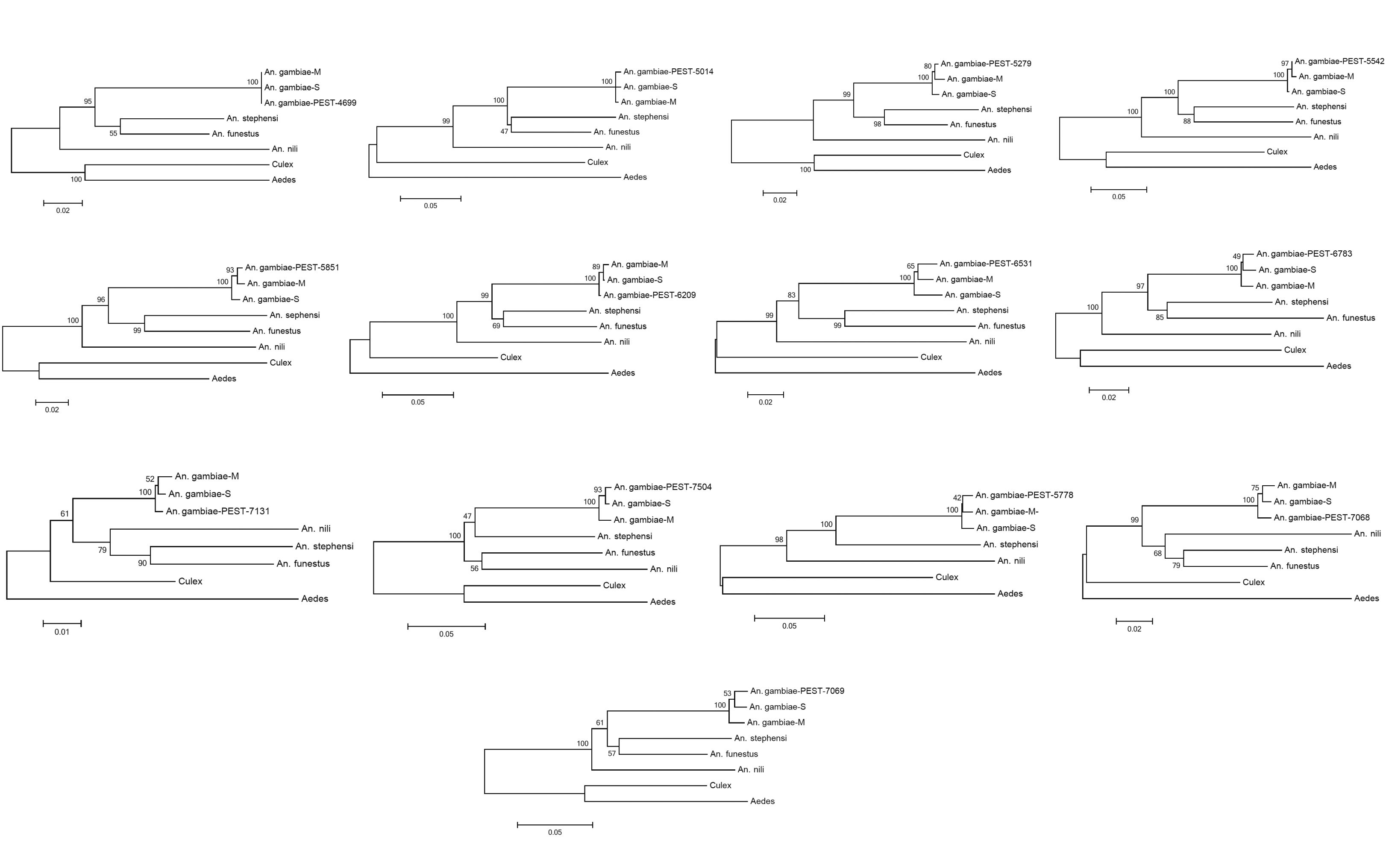

Supplement: Figure S4 — Phylogenetic NJ trees build for AGAP004699, AGAP005014, AGAP005279, AGAP005542, AGAP005851, AGAP006209, AGAP006531, AGAP006783, AGAP007131, AGAP007504, AGAP005778, AGAP007068, and AGAP007069 gene sequences located in the 2L chromosomal arm. Bootstrap values are shown on branches of phylogenetic trees as percentages. (TIF) [file pone.0093580.s004.tif]

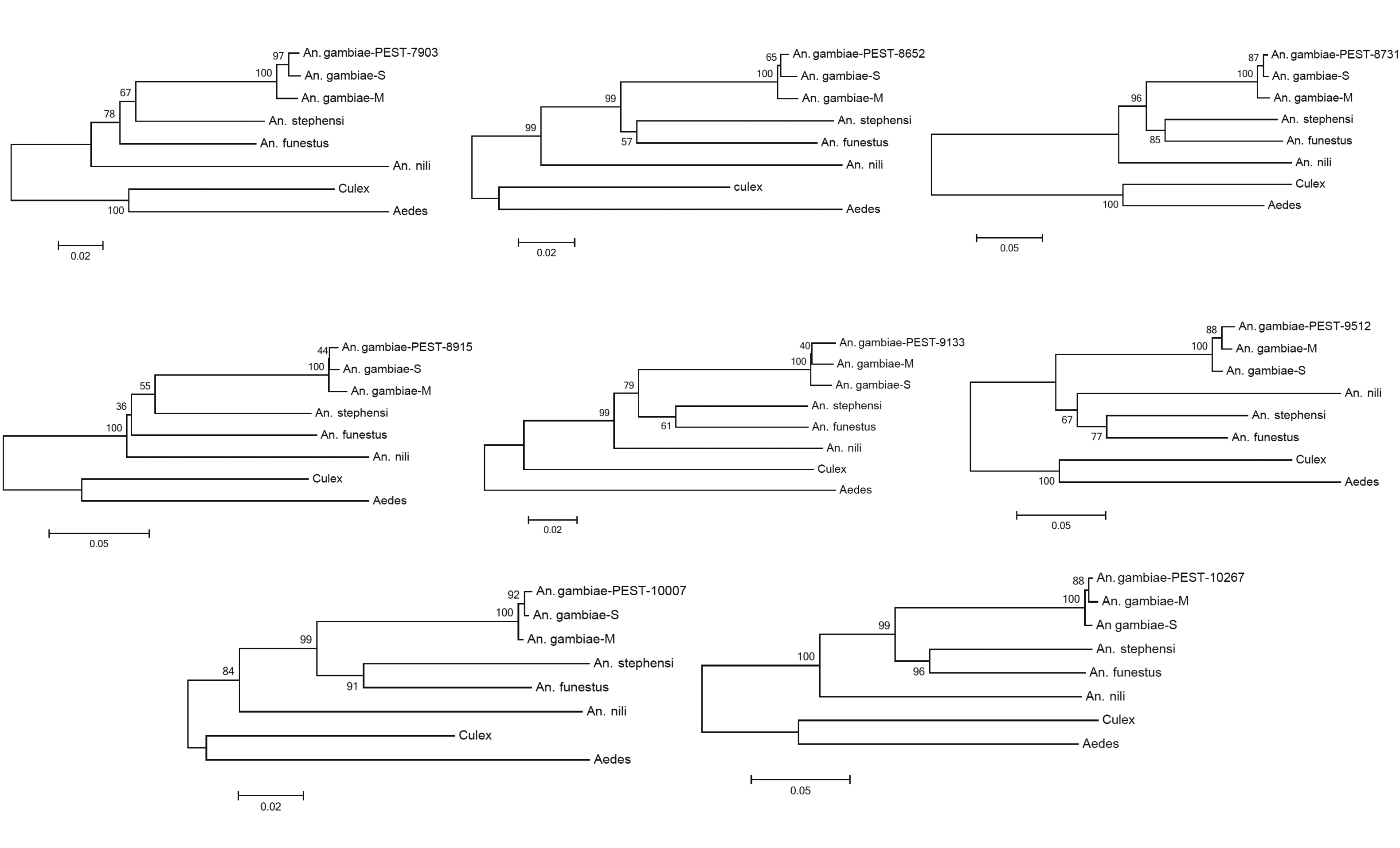

Supplement: Figure S5 — Phylogenetic NJ trees build for AGAP007903, AGAP008652, AGAP008731, AGAP008915, AGAP009133, AGAP009512, AGAP010007, and AGAP010267 gene sequences located in the 3R chromosomal arm. Bootstrap values are shown on branches of phylogenetic trees as percentages. (TIF) [file pone.0093580.s005.tif]

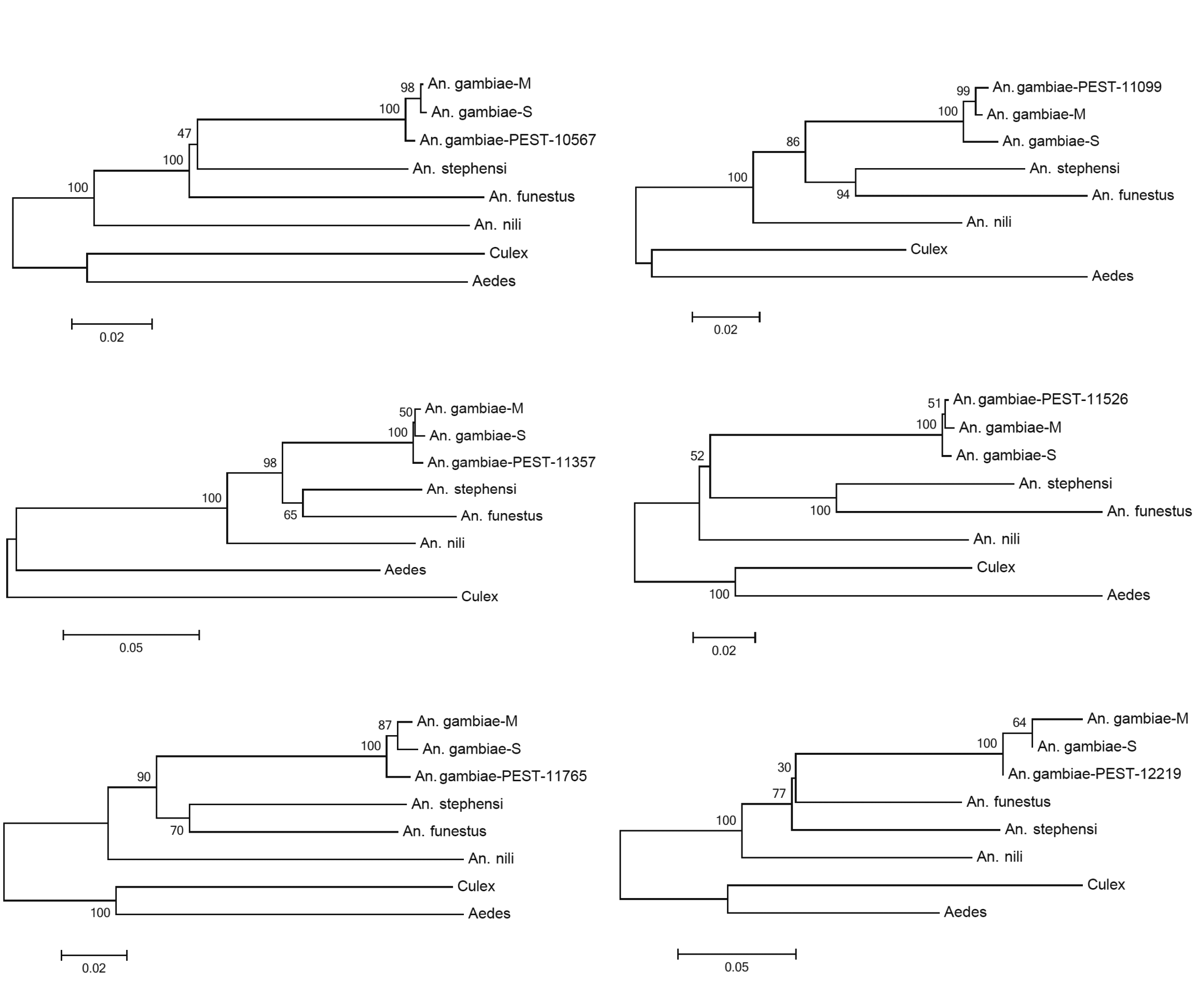

Supplement: Figure S6 — Phylogenetic NJ trees build for AGAP010567, AGAP011099, AGAP011357, AGAP011526, AGAP011765, and AGAP012219 gene sequences located in the 3L chromosomal arm. Bootstrap values are shown on branches of phylogenetic trees as percentages. (TIF) [file pone.0093580.s006.tif]
