# Supplementary material for: Multigene Phylogenetics Reveals Temporal Diversification of Major African Malaria Vectors
Source: PLoS One. 2014 Apr 4;9(4):e93580. doi: 10.1371/journal.pone.0093580 (PMC3976319; doi:10.1371/journal.pone.0093580)
Supplement: Figure S2 — Phylogenetic ML trees build from sequences of 49 genes located in 5 chromosomal arms. Bootstrap values are shown on branches of phylogenetic trees as percentages. (PDF) [file pone.0093580.s002.pdf]

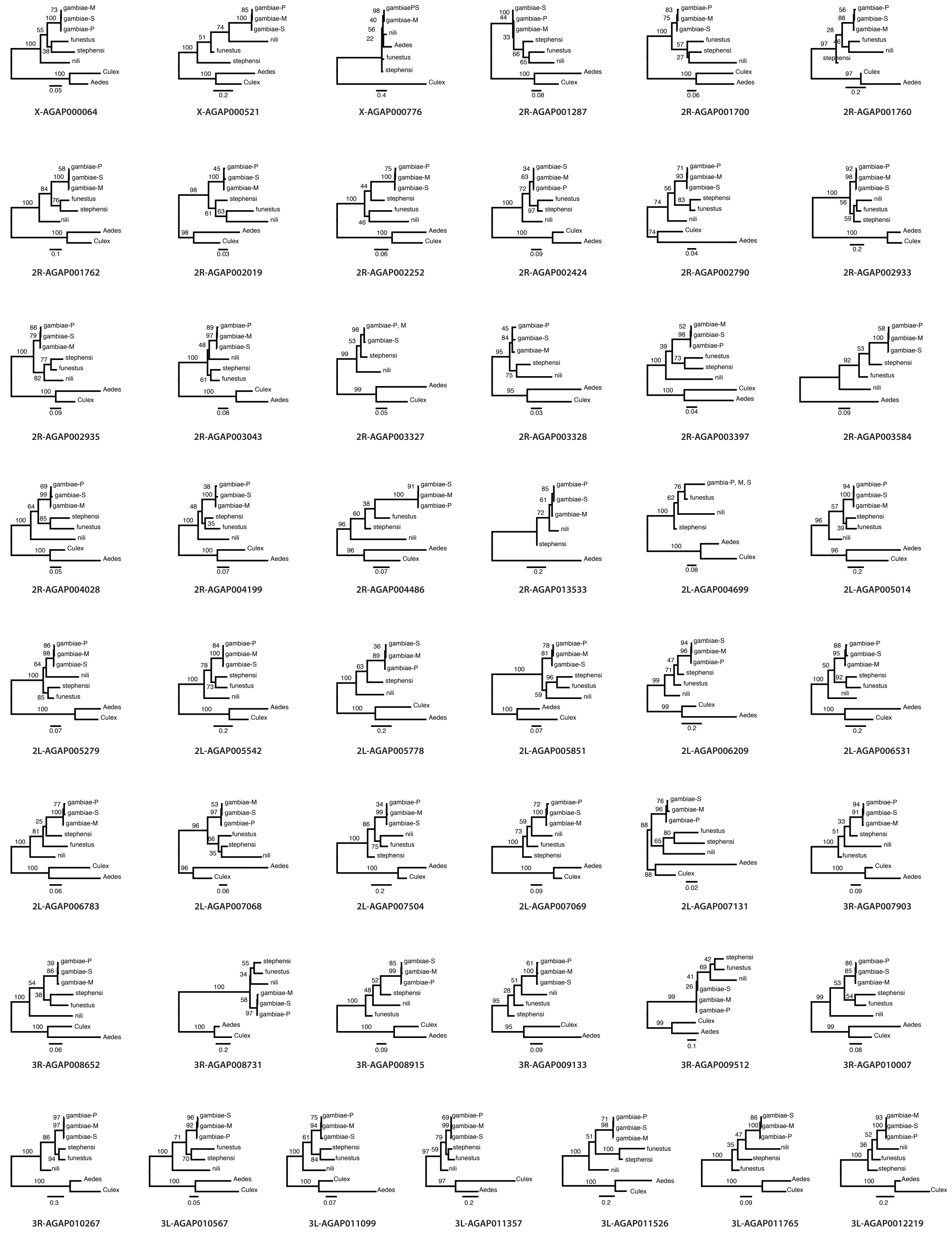
X-AGAP000064
X-AGAP000521
X-AGAP000776
2R-AGAP001287
2R-AGAP001700
2R-AGAP001760
2R-AGAP001762
2R-AGAP002019
2R-AGAP002252
2R-AGAP002424
2R-AGAP002790
2R-AGAP002933
2R-AGAP002935
2R-AGAP003043
2R-AGAP003327
2R-AGAP003328
2R-AGAP003397
2R-AGAP003584
2R-AGAP004028
2R-AGAP004199
2R-AGAP004486
2R-AGAP013533
2L-AGAP004699
2L-AGAP005014
2L-AGAP005279
2L-AGAP005542
2L-AGAP005778
2L-AGAP005851
2L-AGAP006209
2L-AGAP006531
2L-AGAP006783
2L-AGAP007068
2L-AGAP007504
2L-AGAP007069
2L-AGAP007131
3R-AGAP007903
3R-AGAP008652
3R-AGAP008731
3R-AGAP008915
3R-AGAP009133
3R-AGAP009512
3R-AGAP010007
3R-AGAP010267
3L-AGAP010567
3L-AGAP011099
3L-AGAP011357
3L-AGAP011526
3L-AGAP011765
3L-AGAP0012219
