# Supplementary material for: Multigene Phylogenetics Reveals Temporal Diversification of Major African Malaria Vectors
Source: PLoS One. 2014 Apr 4;9(4):e93580. doi: 10.1371/journal.pone.0093580 (PMC3976319; doi:10.1371/journal.pone.0093580)
Supplement: Table S1 — Selected genes from X chromosome and length of orthologous sequences in 6 species. (DOCX) [file pone.0093580.s007.docx]

**Table S1. Selected genes from X chromosome and length of orthologous sequences in 6 species.**

| **X**  **Chromosome** | ***An.***  ***gambiae*-PEST** | ***An. gambiae*-M** | ***An. gambiae*-S** | ***An. stephensi*** | ***An.***  ***nili*** | ***An. funestus*** | ***Aedes*** | ***Culex*** |
| --- | --- | --- | --- | --- | --- | --- | --- | --- |
| AGAP000064 | 774 | 508 | 774 | 772 | 680 | 769 | 597 | 597 |
| AGAP000521 | 1183 | 512 | 655 | 1193 | 661 | 617 | 417 | 420 |
| AGAP000776 | 640* | 593 | 640* | 631 | 619 | 637 | 486 | 252 |

*Asterisks denote identical sequences.
