# Supplementary material for: Multigene Phylogenetics Reveals Temporal Diversification of Major African Malaria Vectors
Source: PLoS One. 2014 Apr 4;9(4):e93580. doi: 10.1371/journal.pone.0093580 (PMC3976319; doi:10.1371/journal.pone.0093580)
Supplement: Table S2 — Selected genes from 2R chromosome and length of orthologous sequences in 6 species. (DOCX) [file pone.0093580.s008.docx]

**Table S2. Selected genes from 2R chromosome and length of orthologous sequences in 6 species.**

| **2R**  **Chromosome** | ***An.***  ***gambiae*-PEST** | ***An. gambiae*-M** | ***An. gambiae*-S** | ***An. stephensi*** | ***An.***  ***nili*** | ***An.***  ***funestus*** | ***Aedes*** | ***Culex*** |
| --- | --- | --- | --- | --- | --- | --- | --- | --- |
| AGAP001287 | 967 | 505 | 505 | 971 | 959 | 493 | 501 | 501 |
| AGAP001700 | 983 | 983 | 983 | 969 | 962 | 482 | 408 | 408 |
| AGAP002019 | 1039 | 1039 | 1039 | 818 | 815 | 808 | 624 | 624 |
| AGAP002252 | 823 | 823 | 823 | 821 | 821 | 440 | 648 | 648 |
| AGAP002424 | 717 | 717 | 711 | 717 | 712 | 380 | 564 | 564 |
| AGAP002790 | 776 | 481 | 481 | 481 | 481 | 475 | 417 | 417 |
| AGAP003043 | 912 | 912 | 913 | 648 | 675 | 633 | 321 | 321 |
| AGAP003397 | 859 | 859 | 859 | 859 | 859 | 798 | 858 | 858 |
| AGAP003584 | 811 | 811 | 811 | 811 | 811 | 580 | 807 | 339 |
| AGAP004028 | 541 | 541 | 541 | 539 | 408 | 487 | 540 | 540 |
| AGAP004199 | 643 | 409 | 643 | 644 | 643 | 639 | 642 | 642 |
| AGAP004486 | 776 | 773 | 773 | 757 | 758 | 695 | 507 | 507 |
| AGAP001760 | 821 | 817 | 821 | 504 | 503 | 502 | 771 | 502 |
| AGAP001762 | 1118 | 707 | 1118 | 1072 | 742 | 335 | 941 | 1100 |
| AGAP002933 | 1086 | 1086 | 1086 | 969 | 895 | 908 | 210 | 207 |
| AGAP002935 | 827 | 826 | 826 | 802 | 486 | 406 | 798 | 1022 |
| AGAP013533 | 618 | 445 | 446 | 235 | 149 | - | 680 | - |
| AGAP003327 | 507* | 507* | 507 | 503 | 505 | 505 | 513 | 508 |
| AGAP003328 | 570 | 569 | 569 | 245 | 243 | - | 132 | 132 |

*Asterisks denote identical sequences.
