# Supplementary material for: Multigene Phylogenetics Reveals Temporal Diversification of Major African Malaria Vectors
Source: PLoS One. 2014 Apr 4;9(4):e93580. doi: 10.1371/journal.pone.0093580 (PMC3976319; doi:10.1371/journal.pone.0093580)
Supplement: Table S3 — Selected genes from 2L chromosome and length of orthologous sequences in 6 species. (DOCX) [file pone.0093580.s009.docx]

Table S3. Selected genes from 2L chromosome and length of orthologous sequences in 6 species.

| **2L**  **Chromosome** | ***An.***  ***gambiae*-PEST** | ***An. gambiae*-M** | ***An. gambiae*-S** | ***An. stephensi*** | ***An.***  ***nili*** | ***An. funestus*** | ***Aedes*** | ***Culex*** |
| --- | --- | --- | --- | --- | --- | --- | --- | --- |
| AGAP004699 | 640* | 640* | 640* | 640 | 639 | 633 | 627 | 582 |
| AGAP005014 | 1400 | 1385 | 1385 | 1187 | 1134 | 510 | 381 | 390 |
| AGAP005279 | 531 | 531 | 531 | 499 | 496 | 484 | 489 | 489 |
| AGAP005542 | 959 | 746 | 959 | 962 | 941 | 894 | 675 | 750 |
| AGAP005851 | 831 | 831 | 831 | 829 | 770 | 770 | 828 | 570 |
| AGAP006209 | 971 | 717 | 717 | 726 | 705 | 706 | 690 | 399 |
| AGAP006531 | 903 | 901 | 900 | 879 | 884 | 457 | 885 | 873 |
| AGAP006783 | 1017 | 1017 | 1016 | 1016 | 1016 | 492 | 1017 | 1017 |
| AGAP007131 | 730 | 730 | 730 | 730 | 730 | 730 | 453 | 456 |
| AGAP007504 | 850 | 850 | 850 | 843 | 851 | 450 | 849 | 849 |
| AGAP005778 | 1216 | 1216 | 1216 | 1208 | 981 | - | 552 | 546 |
| AGAP007068 | 712 | 712 | 712 | 718 | 712 | 539 | 555 | 555 |
| AGAP007069 | 364 | 364 | 364 | 364 | 364 | 293 | 360 | 360 |

*Asterisks denote identical sequences.
