# Supplementary material for: Multigene Phylogenetics Reveals Temporal Diversification of Major African Malaria Vectors
Source: PLoS One. 2014 Apr 4;9(4):e93580. doi: 10.1371/journal.pone.0093580 (PMC3976319; doi:10.1371/journal.pone.0093580)
Supplement: Table S4 — Selected genes from 3R chromosome and length of orthologous sequences in 6 species. (DOCX) [file pone.0093580.s010.docx]

Table S4. Selected genes from 3R chromosome and length of orthologous sequences in 6 species.

| **3R**  **Chromosome** | ***An.***  ***gambiae*-PEST** | ***An. gambiae*-M** | ***An. gambiae*-S** | ***An. stephensi*** | ***An.***  ***nili*** | ***An.***  ***funestus*** | ***Aedes*** | ***Culex*** |
| --- | --- | --- | --- | --- | --- | --- | --- | --- |
| AGAP007903 | 921 | 927 | 927 | 906 | 767 | 650 | 402 | 402 |
| AGAP008652 | 597 | 595 | 596 | 571 | 561 | 575 | 453 | 453 |
| AGAP008731 | 800 | 800 | 800 | 799 | 524 | 542 | 573 | 549 |
| AGAP008915 | 795 | 795 | 795 | 801 | 419 | 599 | 411 | 408 |
| AGAP009133 | 880 | 880 | 880 | 769 | 610 | 612 | 483 | 483 |
| AGAP009512 | 549 | 549 | 549 | 541 | 530 | 353 | 207 | 393 |
| AGAP010007 | 912 | 912 | 912 | 912 | 719 | 821 | 498 | 498 |
| AGAP010267 | 943 | 946 | 943 | 798 | 801 | 796 | 438 | 438 |
