# Supplementary material for: Multigene Phylogenetics Reveals Temporal Diversification of Major African Malaria Vectors
Source: PLoS One. 2014 Apr 4;9(4):e93580. doi: 10.1371/journal.pone.0093580 (PMC3976319; doi:10.1371/journal.pone.0093580)
Supplement: Table S5 — Selected genes from 3L chromosome and length of orthologous sequences in 6 species. (DOCX) [file pone.0093580.s011.docx]

Table S5. Selected genes from 3L chromosome and length of orthologous sequences in 6 species.

| **3L**  **Chromosome** | ***An.***  ***gambiae*-PEST** | ***An. gambiae*-M** | ***An. gambiae*-S** | ***An. stephensi*** | ***An.***  ***nili*** | ***An. funestus*** | ***Aedes*** | ***Culex*** |
| --- | --- | --- | --- | --- | --- | --- | --- | --- |
| AGAP010567 | 1173 | 1173 | 1173 | 1171 | 1173 | 938 | 1170 | 1023 |
| AGAP011099 | 759 | 759 | 759 | 758 | 580 | 497 | 759 | 759 |
| AGAP011357 | 743 | 743 | 743 | 741 | 742 | 741 | 381 | 396 |
| AGAP011526 | 828 | 828 | 828 | 801 | 803 | 401 | 744 | 744 |
| AGAP011765 | 1069 | 1069 | 1069 | 1069 | 1073 | 658 | 843 | 1062 |
| AGAP012219 | 1040 | 607 | 1040 | 1035 | 720 | 1023 | 489 | 489 |
